# Quality Indicators for the Care of People with Intellectual Disability in Family Medicine in Slovenia: A Modified Delphi Study

**DOI:** 10.2478/sjph-2026-0009

**Published:** 2026-06-01

**Authors:** Ana Perdih, Davorina Petek

**Affiliations:** University of Ljubljana, Faculty of Medicine, Department for Family Medicine, Poljanski nasip 58, 1000 Ljubljana, Slovenia; Zdravstveni zavod Revita, Peričeva ulica 31, 1000 Ljubljana, Slovenia; Zdravstveni zavod Zdravje, Vilharjev podhod 1, 1000 Ljubljana, Slovenia

**Keywords:** Intellectual disability, Quality indicators, Delphi technique, Primary care, motnje v duševnem razvoju, kazalniki kakovosti, delfska študija, družinska medicina

## Abstract

**Introduction:**

People with intellectual disability (ID) experience poorer health outcomes and shorter life expectancy than the general population—gaps that could be mitigated by high-quality healthcare. In Slovenia, there are no specific recommendations for this population. The aim of this study was to identify and validate quality indicators (QIs) for the care of people with ID in family medicine, to serve as recommendations for this population in Slovenia.

**Methods:**

A three-round Delphi study was conducted with 15 national experts. An initial set of 44 indicators, derived from the literature, was presented, and panel members proposed eight additional indicators. In the first round, panel members rated the importance of each indicator. Based on feedback, the indicators were revised to separate importance from the implementation interval. In the second and third rounds, panel members rated importance and feasibility and selected the most appropriate time interval.

**Results:**

A total of 33 indicators were validated. These indicators cover multiple aspects of healthcare, including lifestyle factors, lifestyle advice, clinical outcomes, ongoing care, preventive medicine, and administration. Of the original 44 indicators, 28 were confirmed (64%). Of the 8 indicators proposed by panel members, 5 were validated (62.5%). A total of 9 of the original indicators (20%) and 3 of the panel-proposed indicators (37.5%) were considered important but not feasible.

**Conclusions:**

This three-round Delphi study successfully developed QIs for the care of people with ID in Slovenia. These QIs can be integrated directly into existing system structures. The study provides a set of indicators that can inform the development of a clinical checklist and serve as a practical tool for evaluating both organisational and clinical aspects of quality of care.

## Izvleček

**Uvod:**

Osebe z motnjo v duševnem razvoju (MDR) imajo v povprečju slabše zdravstveno stanje in krajšo pričakovano življenjsko dobo kot splošna populacija. Na oboje lahko vplivamo z zagotavljanjem kakovostne zdravstvene oskrbe. V Sloveniji ni priporočil za kakovostno oskrbo oseb z MDR na primarnem nivoju. Namen raziskave je bil razviti in validirati kazalnike, ki bi bili osnova za ugotavljanje kakovosti obravnave oseb z MDR v družinski medicini v Sloveniji.

**Metode:**

V delfski študiji je sodelovalo 15 slovenskih zdravnikov. V prvem krogu raziskave so prejeli 44 predlogov kazalnikov, pripravljenih na podlagi literature, za oceno pomembnosti kazalnika (9-stopenjska Likertova lestvica). V prvem krogu so panelisti dodali še 8 predlogov. Predlagali so, da se ocenjuje posebej vsebina predlaganega kazalnika in frekvenca, s katero naj se kazalnik izvaja. Tako so v drugem krogu ocenjevali vseh 52 kazalnikov glede pomembnosti in izvedljivosti ter izbirali frekvenco. V tretji krog so napredovali kazalniki, ki so dosegli povprečje pomembnosti in izvedljivosti 7 ali več.

**Rezultati:**

Panelisti so potrdili 33 kazalnikov kakovosti. Izmed predlaganih 44 so jih potrdili 28 (64 %). Izmed 8, ki so jih predlagali v prvem krogu, pa so jih potrdili 5 (62,5 %). Za 20 % kazalnikov, predlaganih v prvem krogu (9 kazalnikov), so ocenili, da so pomembni, niso pa izvedljivi v družinski medicini v Sloveniji; enako so ocenili za 37,5 % tistih kazalnikov, ki so jih predlagali sami (3 kazalniki). Potrjeni kazalniki kakovosti zajemajo različna področja zdravstvene obravnave in so razvrščeni v šest sklopov: dejavniki življenjskega sloga (npr. beleženje kajenja, uživanja alkohola, BMI, pogovor o zaprtju), svetovanje o življenjskem slogu (npr. telesna dejavnost, opuščanje kajenja), klinični izidi (npr. merjenje krvnega tlaka, glukoze in TSH, obravnava epilepsije), tekoča obravnava (npr. letni pregled farmakoterapije), administrativni elementi (npr. kontakt skrbnika, socialna anamneza, oseba za podajo soglasja) ter preventivna medicina (npr. vključevanje v presejalne programe in cepljenja). Devet kazalnikov ne zahteva časovnega intervala (zapisi v zdravstveni dokumentaciji), 11 je predvidenih za letni, 13 pa za dvoletni pregled, kar je skladno z obstoječimi poteki dela v družinski medicini.

**Zaključki:**

Delfska študija je potekala v treh krogih, v katerih so panelisti potrdili 33 kazalnikov kakovosti za obravnavo oseb z MDR v družinski medicini v Sloveniji. Dosežen je bil konsenz glede pomembnosti in izvedljivosti kazalnikov ter glede frekvence, ko naj se posamezni kazalnik izvaja. Raziskava je tako privedla do kazalnikov, ki jih je možno neposredno vključiti v delo zdravnikov in diplomiranih sester v ambulantah družinske medicine.

## 1 INTRODUCTION

People with intellectual disability (ID) represent 1–3% of the population, with higher prevalence in low- and middle-income countries (1). Individuals with ID exhibit varying levels of intellectual functioning and adaptive behaviour compared with the general population (2). These differences affect multiple aspects of life, including healthcare – partly due to limited ability to communicate needs (3, 4) and partly due to stigma (5, 6).

In many European countries, most healthcare for people with ID is delivered in primary care (7). In Slovenia, a network of Child Development Units (CDU) provides multidisciplinary care for children with special needs, including ID (8). Children are referred to these units by primary care paediatricians and are followed up there until adulthood.

Some adults with ID enter Centres for Training, Work, and Care (CTWC), which are residential institutions with a general practitioner (GP) and a multidisciplinary team. Others remain at home and visit GPs in community health centres, where multidisciplinary care is not integrated. These people access services through the same system as the general population, resulting in infrequent appointments. As people with ID are dispersed among GPs nationwide, most GPs rarely encounter them and therefore have limited experience in providing care (9, 10). Consequently, Slovenia lacks true experts in this field for clinical practice, research, and education.

Research shows that certain conditions occur more often or at an earlier age in people with ID (e.g. diabetes, hypertension and chronic arthritis) (11). People with ID experience poorer health outcomes (12, 13) and higher standardised mortality ratios than the general population (14). To achieve high quality of care (QoC) (15) for people with ID, some countries have established recommendations and guidelines (7, 16). In Slovenia, such guidelines do not exist.

QoC can be assessed using quality indicators (QIs) (17, 18). QIs should be relevant to health outcomes, measurable, and feasible (19), meaning practical and achievable within the system’s resources and constraints. QIs may therefore vary across countries. Several methods exist for developing QIs, including the Delphi technique. This approach is suitable when there are few recognised clinical experts, as it leverages the collective experience of practitioners familiar with the population and helps ensure that the indicators are relevant and feasible within the existing healthcare system.

The aim of this study was to identify and validate QIs for the care of people with ID in family medicine, to serve as recommendations for this population in Slovenia.

## 2 METHODS

The Delphi method is a systematic approach for developing QIs through expert consensus (18). It is conducted using questionnaires without face-to-face meetings, allowing participants to express their views without peer pressure while considering feedback from other panel members (18, 20, 21). This study applied a modified Delphi technique: no open-ended questions were included in the first round, and the study began with predefined QIs distributed for rating. All indicators proceeded to the second round after modification based on panel member feedback.

Anonymised questionnaires were distributed to experts in three rounds, with feedback provided between rounds. Statements were rated on a 9-point Likert scale to achieve consensus, defined as a mean score ≥ 7. After each round, panel members received aggregated ratings and comments before proceeding.

### 2.1 Panelists

Purposive sampling was used to recruit GPs with expertise in quality or experience in the primary care of people with ID via email. To broaden the panel, a snowball sampling technique was applied. Physicians working in CTWC were identified from a government list and invited by phone or email. Paediatricians were recruited through snowball sampling, with initial CDU invitees forwarding the email invitation to colleagues.

In total, 18 experts agreed to participate: 9 GPs with an interest in ID and healthcare quality, 7 physicians currently or previously employed in CTWC, and 2 paediatricians from CDU. The second and third rounds included invitations to the same panel members who participated in the first round.

### 2.2 Study design

A three-round modified Delphi study was conducted between March and December 2024. The initial draft of QIs was based on Slovenian national guidelines for cardiovascular disease prevention in primary care, the guidelines for primary care quality in ID in other countries, and other literature (7, 16, 19, 22). Indicators were grouped into six categories for clarity: lifestyle factors, lifestyle advice, clinical outcomes, ongoing care, administration, and preventive medicine.

### 2.3 First round

Panelists received an Excel file containing 44 proposed indicators and rated their importance using a 9-point Likert scale (“For each indicator, specify how important it is for ensuring good quality healthcare for a person with ID”). Each indicator included a rationale (e.g., “Foreign guidelines include this as part of annual check-up” or “Smoking prevalence is very high in this population”). Participants could comment on their rating and suggest alternative indicators.

### 2.4 Second round

Panel members received the revised list of indicators, including the 8 new suggestions, together with first-round summary statistics (mean, median, distribution) and comments. They rated importance and feasibility (1–9) (“Is it feasible to obtain this indicator in family medicine clinics in Slovenia?”) and selected the most appropriate time interval from 3 options.

### 2.5 Third round

Indicators with a second-round importance mean ≥ 7 advanced to round three. Panel members received feasibility ratings, comments, and time-interval votes, then re-rated feasibility (1–9) and confirmed the time frame interval.

### 2.6 Data collection

Panel members were invited by phone or email and provided consent during initial contact. All subsequent communication (questionnaire distribution, responses, and reminders) was conducted via email. Questionnaires were anonymised by assigning numbers for file naming before return.

### 2.7 Statistical analysis

**Figure 1.**
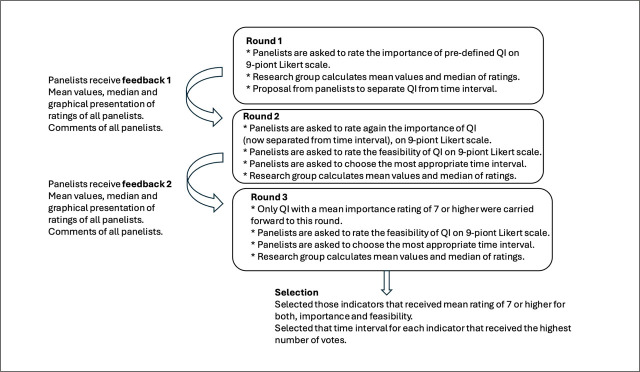
Summary of the study process.

After the first round, mean and median scores were calculated. Due to methodological changes (separating importance from time interval), all indicators proceeded to round two. After round two, mean and median values for importance and feasibility were calculated. In round three, indicators with feasibility ≥ 7 were validated. The time interval with the highest agreement (most votes) was adopted. Figure 1 summarises the study process.

## 3 RESULTS

Of the 18 experts who agreed to participate, 15 panel members (83%) responded in the first round. One family doctor and one CTWC physician did not respond despite reminders; one CDU paediatrician withdrew after receiving the questionnaire, citing insufficient expertise in adult QIs. The remaining 15 panel members completed all rounds. Table 1 presents the characteristics of the panel members.

**Table 1.** Characteristics of panel members.

**No.**	**Gender**	**Specialty**	**Experience**	**Area of expertise**	**Region in Slovenia**
**1**	F	GP/FM	E	Experience in CTWC	Posavska
**2**	F	GP/FM	L	Working in CTWC	Koroška
**3**	F	GP/FM	M	Working in CTWC	Osrednjeslovenska
**4**	F	GP/FM	E	Working in CTWC	Osrednjeslovenska
**5**	M	GP/FM	E	Working in CTWC	Primorska
**6**	F	GP/FM	M	Experience in CTWC	Osrednjeslovenska
**7**	F	paediatrician	L	Working in CDU	Osrednjeslovenska
**8**	F	GP/FM	M	Quality assurance	Osrednjeslovenska
**9**	F	GP/FM	L	Quality assurance	Osrednjeslovenska
**10**	F	GP/FM	L	Quality assurance	Osrednjeslovenska
**11**	F	GP, emergency medicine specialist	M	Quality assurance	Osrednjeslovenska
**12**	M	GP/FM	M	Quality assurance	Osrednjeslovenska
**13**	F	GP/FM	L	Quality assurance	Osrednjeslovenska
**14**	M	GP/FM	M	Quality assurance	Gorenjska
**15**	M	GP/FM	L	Quality assurance	Zasavska

Legend: GP/FM = specialist in general practice/family medicine; E = Early career = first 5 years after specialty exam; M = Middle career = between early and late career; L = Late career = last 10 years before retirement; CDU = Child Development Unit; CTWC = Centres for Training, Work and Care

In the first round, proposed indicators were combined: a QI and a time interval for implementation in practice (e.g. measuring blood pressure every 24 months). Panel members noted that these should be rated separately, and the indicators were therefore redefined for round two. Eight new indicators were suggested in the first round.

At the end of the third round, 33 QIs were validated.

Of the 44 indicators proposed by the researcher, 28 were validated (64%). Seven were classified as not important (16%), and 9 as important but not feasible (20%).

Of the 8 indicators suggested by panel members, 5 were validated (62.5%). None were classified as not important; 3 were considered important but not feasible (37.5%).

### 3.1 Lifestyle factors

Four of the 5 indicators were validated (80%), covering smoking, alcohol consumption, BMI, and discussions on constipation.

### 3.2 Lifestyle advice

Three of the 5 indicators (60%) were validated, covering physical activity, smoking, and alcohol consumption. Two indicators, relating to dental visits and healthy eating, were rated as not feasible.

### 3.3 Clinical outcomes

A total of 11 indicators were validated, including 1 added in the first round. These addressed screening, blood pressure, blood glucose and TSH measurement, specialist referrals, and epilepsy medication. Three indicators, relating to hearing loss and osteoporosis screening, were classified as important but not feasible.

### 3.4 Ongoing care

Three indicators were validated, including 2 added in the first round, relating to pharmacotherapy, social inclusion, and chronic disease follow-up. Six indicators (2 of which were suggested by panel members) were deemed as not feasible, including over-the-counter medication, incontinence, depression, abuse, rehabilitation, and caregiver burnout.

### 3.5 Administration

All 3 proposed indicators were validated, covering contact, social history, and the person responsible for consent.

### 3.6 Preventive medicine

Nine of the 10 indicators were validated, covering inclusion in screening programmes, registered nurse programmes, and vaccinations. The only indicator rated as not feasible was HPV vaccination for sexually active people.

### 3.7 Time intervals

Of the 33 validated indicators, 9 have no time interval, as they represent documentation requirements (e.g., caregiver contact, social integration, vaccination status). The remaining indicators were divided as follows: 11 should be reviewed annually and 13 biennially.

The complete list of validated QIs with time intervals is provided in Table 2.

**Table 2.** List of validated quality indicators with appropriate time intervals.

**A note in the documentation**	

1.	Record whether the person is involved in any form of social integration (specify type).
2.	Record parameters for chronic diseases as monitored.
3.	Record contact details of a relative or caregiver.
4.	Record of the person responsible for decision-making if the person with ID is (or will be) unable to give consent.
5.	Record inclusion in a registered nurse programme (»referenčna ambulanta«).
6.	Record tetanus vaccination in the last 10 years (or refusal).
7.	Record pneumococcal vaccination in accordance with national recommendations (or refusal).
8.	Record vaccination against Haemophilus influenzae and hepatitis B (or refusal).
9.	Record vaccination against tick-borne meningoencephalitis in accordance with national recommendations (or refusal).

**Every 12 months**	

**LIFESTYLE FACTORS**	
1.	Record regular monitoring of digestion, including discussion of constipation.
**LIFESTYLE ADVICE**	
1.	Record a discussion on physical activity.
2.	For smokers, record a discussion on smoking cessation.
3.	For non-smokers, record a discussion on the harmful effects of smoking.
4.	Record a discussion on the harmful effects of alcohol consumption.
**CLINICAL OUTCOMES**	
1.	For people aged over 16 years with no chronic medical conditions: record blood pressure measurement.
2.	For people with a chronic medical condition: record blood pressure measurement.
3.	Record blood glucose measurement.
4.	For people with epilepsy: record a discussion on medication uses and measures required during and after a seizure.
5.	For people taking psychotropic drugs: record referral to a neurologist to assess the justification of therapy.
**ONGOING CARE**	
1.	Record regular review of drug therapy, including indications, dosage, frequency, and adherence.
**PREVENTIVE MEDICINE**	
1.	Record vaccination against seasonal influenza (or refusal).

**Every 24 months**	

**LIFESTYLE FACTORS**	
1.	Record smoking status for people aged over 16 years.
2.	Record alcohol consumption status for people aged over 16 years with no chronic condition.
3.	Record body mass, BMI, or waist circumference for people aged over 16 years.
**CLINICAL OUTCOMES**	
1.	Record a preventive check-up for all people aged over 16 years.
2.	Record TSH level.
3.	For people with Down syndrome or risk constellations: record TSH level.
4.	For people aged over 40 years: record referral to an ophthalmologist (to assess vision and rule out glaucoma).
5.	For people with Down syndrome aged over 30 years: record a referral to an ophthalmologist (to assess vision and rule out glaucoma).
6.	Record referral to a neurologist for any person with ID who has epilepsy.
**ADMINISTRATION**	
1.	Record updated social history: place of residence, caregiver, income?
**PREVENTIVE MEDICINE**	
1.	Record participation (or non-participation) in the Dora1 screening programme and follow-up actions where there is no response.
2.	Record participation (or non-participation) in the Zora2 screening programme and follow-up actions where there is no response.
3.	Record participation (or non-participation) in the Svit3 screening programme and follow-up actions where there is no response.

Legend:

1 Dora: Slovenian national breast cancer screening programme;

2 Zora: Slovenian national cervical cancer screening programme;

3 Svit: Slovenian national colorectal screening programme

## 4 DISCUSSION

### 4.1 Main findings

The study presents the first set of family medicine QIs for people with ID in Slovenia, developed and validated through expert consensus. These indicators cover multiple domains: lifestyle factors, lifestyle advice, clinical outcomes, ongoing care, preventive medicine, and administration. The findings are consistent with recommendations reported in the literature.

### 4.2 Comparison with literature

**Compared with QIs for primary care in the general** population without chronic disease in Slovenia (23), the indicators validated for people with ID in Slovenia are more demanding, requiring earlier initiation and more frequent preventive measures (see Table 3). The only QI in this group that was not validated was osteoporosis screening. Two indicators on osteoporosis screening were proposed, but neither was validated. However, screening using the FRAX index is part of the standard registered nurse programme.

A search of the PubMed database identified only 2 sets of recommendations for primary care for people with ID: from Canada (7) and New South Wales (16). These served as the basis for proposed QIs in this study. Comparison of these with the QIs validated for Slovenia shows common domains across all three: comprehensive health assessment, informed consent, medication review, mental health, physical activity, immunisations, epilepsy, vision screening, and thyroid disease.

This study confirmed the importance of assessing constipation and social inclusion, which are not included in the other two sets of recommendations. Conversely, some of the QIs validated for Slovenia appear in only one of the two external recommendations (e.g. screening programmes, smoking, alcohol, contact, and social history).

However, this study did not validate indicators related to genetics, nutritional risk, *H. pylori* screening, or dementia. Although panel members considered these important, they rated several features from the other two recommendations as not feasible (e.g. oral health, hearing, osteoporosis, HPV immunisation). A detailed comparison of all three sources is provided in Table 4.

**Table 3.** Comparison of quality indicators (QIs) for primary care in the general population without chronic disease in Slovenia and QIs for people with intellectual disability (ID) in Slovenia.

**Quality indicator**	**General population in Slovenia**	**People with ID in Slovenia**
**Smoking**	Check every 5 years for people aged over 30 years.	Check every 2 years for people aged over 16 years.
		For smokers, record a discussion on smoking cessation annually.
		For non-smokers, record a discussion on the harmful effects of smoking annually.
**Alcohol consumption**	Check every 5 years for people aged over 30 years.	Check every 2 years for people aged over 16 years.
		For people with ID, record a discussion on the harmful effects of alcohol consumption annually.
**Tetanus vaccination**	Regular revaccination against tetanus.	Record tetanus vaccination within the last 10 years (or refusal).
**Cardiovascular risk assessment**	Every 5 years for people aged over 30 years.	Every 24 months, perform a preventive check-up for people with ID aged over 16 years.
**FRAX index**	Every 5 years.	/
**Chronic diseases**	Maintain a list of chronic diagnoses.	Monitor parameters for chronic diseases appropriately.

**Table 4.** Comparison of QIs in international recommendations and QIs validated for people with ID in Slovenia.

**QI**	**Canada**	**New South Wales**	**Slovenia**
**Oral health**	Dentist every 6 months	Dentist annually	Rated important but not feasible
**Vision**	Refer once before 40 years (30 years for DS), then every 2 years	Every 5 years after 45 years (30 years for DS)	Every 2 years after 40 years (30 years for DS)
**Hearing**	Every 5 years after 45 years (3 years for DS)	Every 5 years after 45 years (3 years for DS)	Rated important but not feasible
**Nutrition risk**	Annual counselling	Annually	Rated not important
**Constipation**	/	“Treat proactively”	Record discussion annually
**Epilepsy**	Regular reassessment; education	Record frequency, therapy, and side effects annually; neurologist referral annually	Record medication and seizure behaviour annually; neurologist referral every 24 months
**Thyroid**	Screen every 1–3 years (more often for DS)	Every 3–5 years (annually for DS)	TSH every 24 months
**GERD/*H. pylori***	Screen GERD; test for *H. pylori* if symptomatic	Emphasised, without specific recommendations	Testing for *H. pylori* rated not important
**Osteoporosis**	Screen high-risk from age 19 years	Annual Vit D and Ca	Screening rated important but not feasible; Vit D/Ca not important
**Immunisations**	Hib and pneumococcus	NHMRC guidelines; hep A/B; annual influenza; every 5 years for pneumococcus for people with chronic diseases	Record vaccinations/refusal; H. influenzae and hepatitis B; tetanus every 10 years; pneumococcus and TBE per national recommendations; influenza annually
**Screening programmes**		Annually review of screening participation	Record inclusion in Dora, Zora, Svit every 2 years
**Medication review**	Every 3 months	Every 3–6 months	Review annually.
**Physical activity**	Promote healthy living	30 min/day most days	Discussion annually; BMI/waist every 24 months
**Genetics**	Refer if cause unknown	Consider in adults without a diagnosis	/
**Women’s health**		As for the general population; annual review of menstrual history / menopause symptoms	
**STDs/abuse**	Screen sexual practices; education	/	HPV immunisation rated important but not feasible
**Smoking/alcohol**	/	Annual review and advice	Status every 2 years; annual discussion on harmful effects
**Mental health, difficult behaviours**	Assess levels of adaptive functioning	Assess for underlying physical or external factors in behavioural change	Annual neurologist referral for psychotropic drugs; depression screening rated important but not feasible
	Justify psychotropic drug use annually		
	When behavioural problems or a psychiatric diagnosis, assess possible physical, environmental, and emotional factors		
**Dementia**	Baseline assessment at 40 years	Emphasised, without specific recommendations	Screening after 40 years rated not important
**Comprehensive assessment**	Screen CVD earlier	Annually	Inclusion in a registered nurse programme; preventive check every 2 years; annual BP and glucose
**Contact**		Record guardian and social history annually	Record caregiver contact details; social history every 2 years
**Abuse/neglect**	Screen annually for abuse/neglect and report		Rated important but not feasible
**Informed consent**	Encourage advance planning; assign legal substitute decision-makers	Identify responsible person annually	Record responsible person
**Social inclusion**			Record inclusion

Legend: DS = Down syndrome; GERD = Gastroesophageal reflux disease; TBE = Tick-borne encephalitis; STD = Sexually transmitted disease; CVD = Cardiovascular disease; NHMRC = National Health and Medical Research Council

### 4.3 Outcomes

Validated QIs span a wide range, from organisational aspects to specific clinical measures. Some indicators are specific to this population (e.g. caregiver contact, identification of the person responsible for decision-making), while others apply to the general population but are not considered quality measures in that context (e.g. inclusion in a registered nurse programme, discussion of constipation).

Validated QIs should be reviewed regularly, either annually or biennially. This is feasible, given that annual examinations for people with chronic diseases are already standard in Slovenian family medicine (23). Although each QI was rated individually as feasible, it is uncertain whether all can be addressed within routine appointments, particularly given time constraints. Registered nurses, who form an integral part of family medicine teams in Slovenia (24, 25), could take responsibility for many of these indicators.

Four indicators related to smoking and alcohol use revealed inconsistencies. Panel members discussed the appropriate frequency, noting variation by age and habits. They agreed to discuss harmful effects annually but to record status only biennially. As this approach lacks consistency, both are recommended annually.

### 4.4 Strengths and limitations

#### 4.4.1 Strengths

Panel members were recruited from three distinct areas of care for people with ID, providing a broad range of experience. All were part of the Slovenian healthcare system, ensuring familiarity with what is feasible in practice. Panel members also represented several geographical regions, which is relevant given documented interregional differences in morbidity and mortality (26).

#### 4.4.2 Limitations

The Delphi method has inherent limitations, including the lack of a standardised definition of consensus (20). Panel members were selected for their expertise rather than as a representative sample. As Slovenia lacks comprehensive training in this area, participants were not traditional “experts” but clinicians most engaged in, and experienced in, the care of people with ID in clinical practice.

We modified the Delphi technique in two ways: first, by predefining proposed QIs; second, by allowing all indicators to proceed to the second round after separating QIs from time intervals based on panel member feedback. Skipping the open-ended first round may have limited the emergence of novel indicators and introduced bias towards existing recommendations, although panel members were still invited to suggest additional indicators. We provided a brief rationale for each proposed indicator in the first Delphi round to ensure a shared understanding among panel members. Some participants were quality-of-care experts with limited experience in working with people with ID; the rationales were therefore intended to support more informed judgments. While this context helped to broaden perspectives, it may also have introduced a framing effect and reduced the diversity of initial opinions, as well as the range of additional indicators suggested by panel members.

### 4.5 Feasibility

Of the proposed QIs, 23.1% were considered important but not feasible. Some limitations stem from the Slovenian healthcare system (e.g. dental visits), while others relate to a lack of insurance coverage (e.g. osteoporosis screening). Additional barriers are likely to relate to stigma (27) or limited understanding of people with ID. Panel members noted that, while HPV vaccination and screening for depression, incontinence, caregiver burnout, and abuse were important, they were not feasible. The study did not investigate further why these elements were perceived as not feasible. However, the literature identifies time constraints, communication challenges, and limited experience in addressing these specific issues in people with ID as the most frequent limiting factors (28,29,30).

This study aimed to validate QIs for assessing QoC for people with ID. However, QoC evaluation involves more than adherence to QIs; other dimensions include accessibility, patient-centeredness, and perceived quality from the user’s perspective (31). In this study, the outcome is a list of measures to be performed. The Canadian and Australian guidelines (7, 16) provide recommendations and guidance with explanatory content that supports understanding of this vulnerable population. The QIs can serve as a tool for assessing QoC but are unlikely to shift clinicians’ perspectives or significantly impact overall care. Therefore, a ready-to-use clinical checklist should be developed based on the QIs defined by this study and piloted in a clinical setting.

## 5 CONCLUSION

Fifteen panel members shared their expert opinions in a modified Delphi process to validate QIs for the care of people with ID in family medicine in Slovenia. A total of 33 QIs were validated across six domains: lifestyle factors, lifestyle advice, clinical outcomes, ongoing care, preventive medicine, and administration. Consensus was reached on their importance, feasibility, and the appropriate time intervals for each indicator. These QIs can be integrated directly into existing system structures, particularly through the activities of registered nurse programmes in Slovenian family medicine. The study therefore provides a set of indicators that can inform the development of a clinical checklist and serve as a practical tool for evaluating both organisational and clinical aspects of QoC. Future research should assess how implementation within these structures influences health outcomes and should include pilot testing in selected practices to evaluate usability and workload implications.
